# Effects of grain size and small-scale bedform architecture on CO_2_ saturation from buoyancy-driven flow

**DOI:** 10.1038/s41598-023-29360-y

**Published:** 2023-02-11

**Authors:** Hailun Ni, Sahar Bakhshian, T. A. Meckel

**Affiliations:** Bureau of Economic Geology, Jackson School of Geosciences, The University of Texas at Austin, Austin, TX 78758 USA

**Keywords:** Carbon capture and storage, Hydrology

## Abstract

Small-scale (mm-dm scale) heterogeneity has been shown to significantly impact CO_2_ migration and trapping. To investigate how and why different aspects of small-scale heterogeneity affect the amount of capillary trapping during buoyancy-driven upward migration of CO_2_, we conducted modified invasion percolation simulations on heterogeneous domains. Realistic simulation domains are constructed by varying two important aspects of small-scale geologic heterogeneity: sedimentary bedform architecture and grain size contrast between the matrix and the laminae facies. Buoyancy-driven flow simulation runs cover 59 bedform architecture and 40 grain size contrast cases. Simulation results show that the domain effective CO_2_ saturation is strongly affected by both grain size and bedform architecture. At high grain size contrasts, bedforms with continuous ripple lamination at the cm scale tend to retain higher CO_2_ saturation than bedforms with discontinuous or cross lamination. In addition, the “extremely well sorted” grain sorting cases tend to have lower CO_2_ saturation than expected for cross-laminated domains. Finally, both a denser CO_2_ phase and greater interfacial tension increase CO_2_ saturation. Again, variation in fluid properties seems to have a greater effect on CO_2_ saturation for cross-laminated domains. This result suggests that differences in bedform architecture can impact how CO_2_ saturation values respond to other variables such as grain sorting and fluid properties.

## Introduction

CO_2_ geologic storage, or the injection and sequestration of captured CO_2_ in deep geologic formations such as saline aquifers, is an imperative measure to address climate change^1–4^. Prior research has shown that even small-scale (mm-dm scale) geologic heterogeneity can greatly affect CO_2_ flow and trapping^5–21^. Depositional laminations and baffles are examples of such small-scale heterogeneity, and they have been shown to form effective capillary barriers that can retain a substantial amount of above-residual CO_2_ saturation during both the injection (drainage) and the post-injection (imbibition) stages through the mechanism known as local capillary trapping (LCT), also called capillary heterogeneity trapping^5,8,16,22^. Hence, small-scale heterogeneity can greatly impact how much CO_2_ is retained in the geologic material (the storage capacity of the reservoir) and it is also crucial in controlling the CO_2_ plume migration speed and extent^16,19,20,23,24^. Therefore, it is important to conduct simulations that are capable of correctly incorporating this extra amount of CO_2_ residual or capillary trapping in order to accurately predict how the CO_2_ plume migrates through heterogeneous domains.

Conventional reservoir simulations used to study CO_2_ plume migration and trapping employ coarse (10–100 m scale) grid blocks or cells greatly above the resolution of small-scale heterogeneity to save computational time and resources, but consequently run the risk of obtaining inaccurate simulation results without proper upscaling^16,19,20^. Furthermore, conventional full-physics simulators use continuum-scale Darcy-flow physics and have convergence issues modeling low-rate CO_2_ flow through highly heterogeneous domains when LCT is incorporated^25^. On the other hand, the modified invasion percolation (MIP) method can easily handle large, high-resolution, heterogeneous domains with LCT effects thanks to its simplified physics^25–27^. Invasion percolation (IP) simulation methods originated from simulating multiphase fluid flow in pore networks. MIP methods extend the IP algorithm to the continuum scale, allowing the usage of continuum-scale grid properties (porosity, threshold capillary pressure, etc.) and also include gravity forces^28–31^. MIP simulators significantly reduce the complexity of the fluid flow physics by assuming viscous forces to be negligible, so they can run several orders of magnitude faster than full-physics simulators^26,27^.

The MIP method can be applied to simulate CO_2_ geologic storage because capillary and gravity forces strongly dominate the vast majority of the plume over the entire post-injection time period, which can last for hundreds of years or longer^19,32,33^. Under such flow regimes that are most relevant to CO_2_ geologic storage, the impact of small-scale heterogeneity is especially pronounced due to the lack of viscous forces^22,25,34^. Hence, the ability to correctly quantify the resulting LCT is highly important in accurately predicting plume migration and storage capacity for CO_2_ geologic storage^16,19,20,23,35^. Therefore, in this study, we run MIP simulations on fine-grid heterogeneous domains to easily incorporate the effect of LCT and obtain the domain effective CO_2_ buoyant flow saturation value, which is highly heterogeneity dependent.

**Figure 1:**
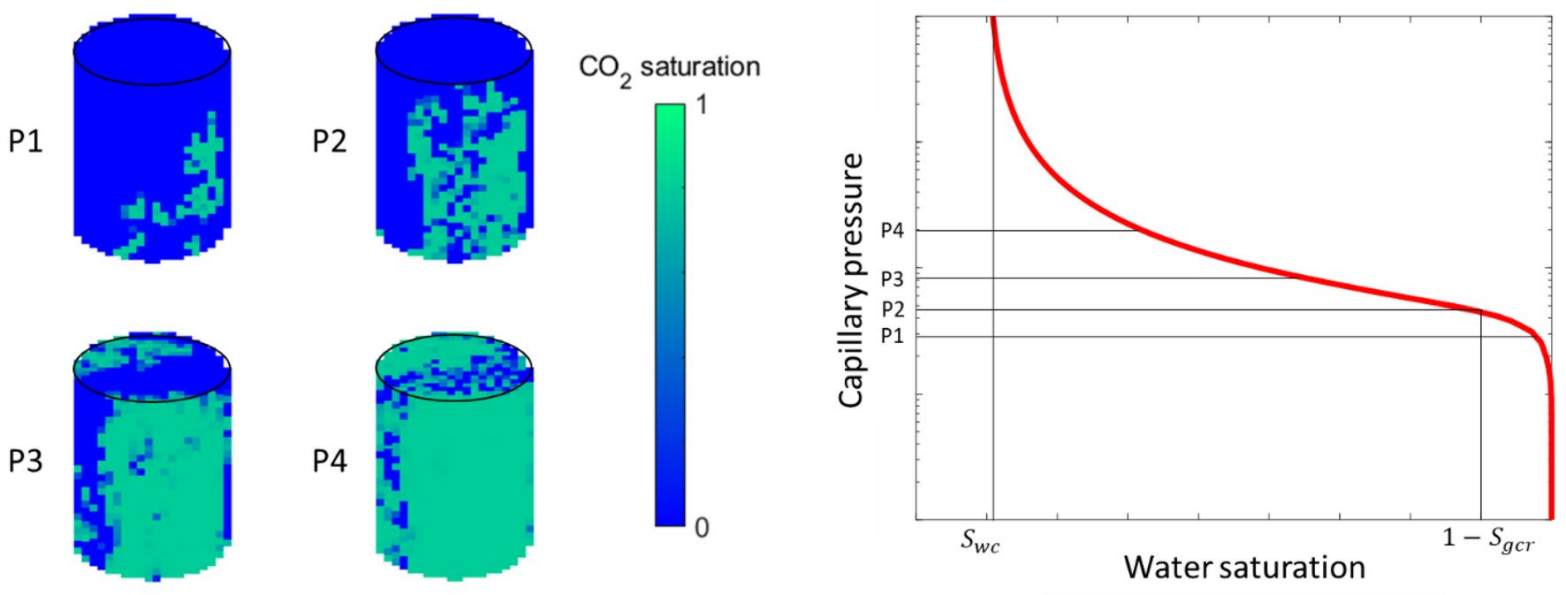
Illustration of buoyancy-driven CO_2_ invading a water-saturated core from the bottom. Left: CO_2_ saturation fields at increasing capillary pressure values. Right: The resulting drainage capillary pressure curve. ${S}_{wc}$: Irreducible water saturation. ${S}_{gcr}$: Critical CO_2_ saturation. P2: Threshold capillary pressure.

This domain effective CO_2_ buoyant flow saturation is the saturation at which buoyancy-driven CO_2_ breaks through or percolates the fine-grid domain during primary drainage. It is therefore also the critical CO_2_ saturation for the equivalent upscaled coarse cell at which CO_2_ begins to form a continuous phase in the current cell and can start flowing to the next cell. For the coarse cell, this is the lowest nonzero CO_2_ saturation point (critical saturation) on a CO_2_ relative permeability curve and corresponds to the threshold capillary pressure (P2) value on the drainage capillary pressure curve as shown in Fig. 1. The ability to correctly assign critical CO_2_ saturation values to coarse field-scale simulation cells is essential, as critical CO_2_ saturation directly affects the estimated CO_2_ dynamic storage capacity. For example, using a flume tank deltaic geologic model, field-scale MIP simulation results obtained on a high-resolution heterogeneous domain demonstrate that by varying the critical CO_2_ saturation from 3 to 48% in the cells, the total amount of CO_2_ retained in the system is doubled^23,36^. Although the critical CO_2_ saturation is located on the drainage capillary pressure curve, if imbibition occurs at this point, the final residual CO_2_ saturation retained is still quite close to the original critical CO_2_ saturation. Previous tank-scale beadpack experimental results show that 74–89% of the drainage critical CO_2_ saturation is residually trapped after spontaneous imbibition^22,37^. Therefore, the critical CO_2_ saturation provides a close upper limit on post-imbibition CO_2_ residual trapping resulting from buoyancy-driven flow.

The goal of this work is to conduct MIP numerical fluid flow simulations to explore how different types and degrees of small-scale capillary heterogeneity affect CO_2_ buoyant flow saturation in the context of realistic sedimentary bedform architectures and matrix/laminae grain size contrasts. This work builds upon a previous study by Trevisan et al. in which MIP simulations were conducted on eight three-dimensional (3D) domains with realistic sedimentary bedforms^18^. Trevisan et al. discovered the strong dependence of CO_2_ saturation on both grain size and bedform architecture. CO_2_ saturation is found to increase nonlinearly with grain size contrast in a predictive manner, forming a distinct S-shaped curve. It is also found that grain sorting has an impact on how well the CO_2_ saturation values conform to the fitted S curve. However, with just eight bedforms, the limited size of the simulation results hinders deeper understanding. Therefore, the novelty of the current work is that it not only significantly expands the simulation dataset compared to the previous study (from 8 to 59 bedforms), but it also explains how the difference in small-scale bedform architecture (ripple vs. cross-lamination) affects CO_2_ saturation at different grain size contrast, grain sorting, and fluid property values.

## Simulation methods

### Simulation domains

**Figure 2:**
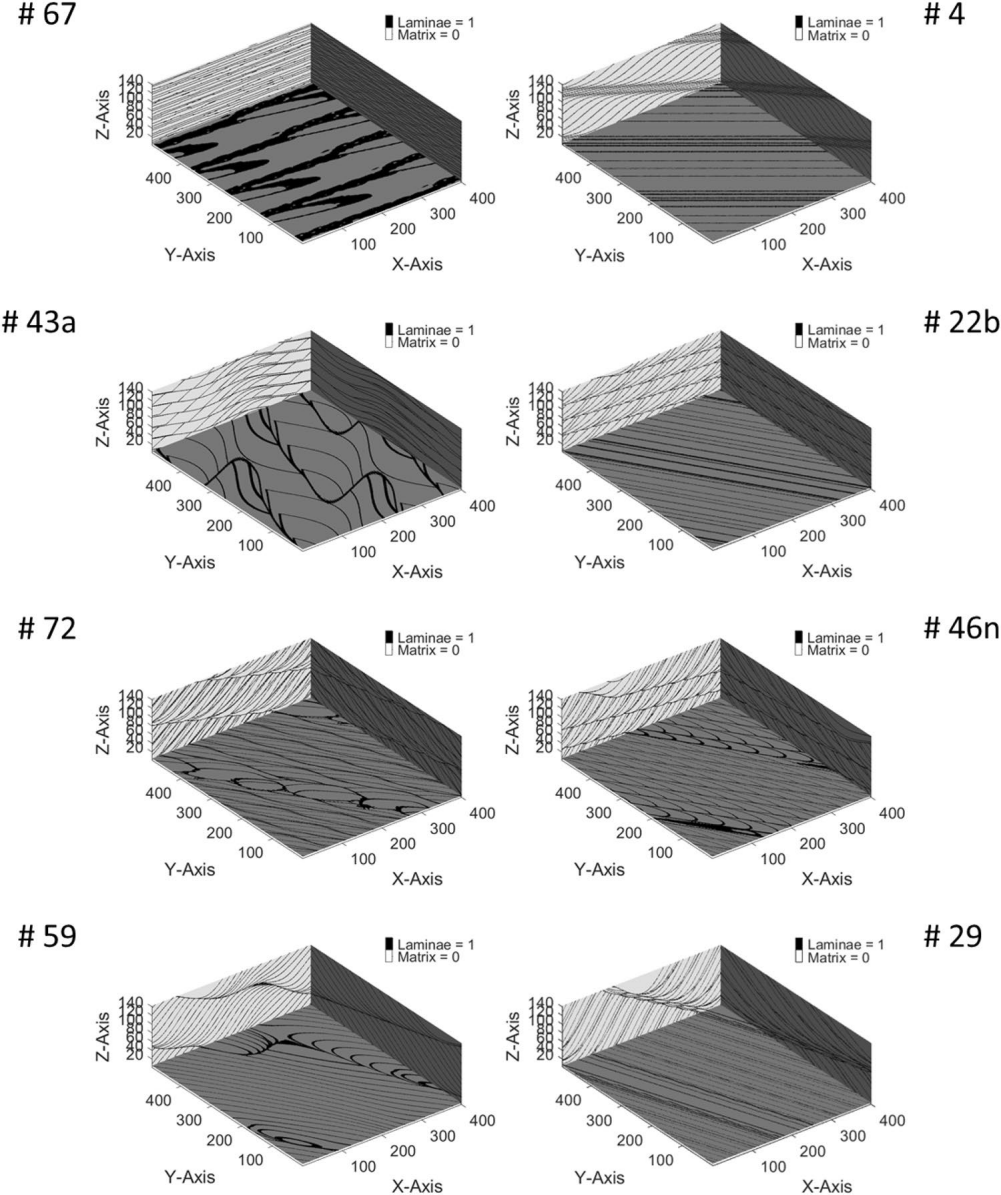
Visualization of eight selected 3D two-facies BAMs^38–40^.

Rubin and Carter^38^ have previously compiled a series of realistic bedform architecture models (BAMs). Each BAM consists of a coarse-grained matrix facies and a fine-grained laminae facies. The BAMs used in the study represent a wide variety of cm- to dm-scale heterogeneity patterns seen in sandstone formations resulting from different depositional environments. This study applies the same simulation methods used by Trevisan et al.^18^ to 59 of the 62 available BAMs. Note that three BAMs (#8, #9, and #10) are excluded from this study because they do not have lamination structures within them. Some example BAMs are shown in Fig. 2. BAM #4 is formed by “two-dimensional, stoss-depositional bedforms climbing at a subvertical angle”. BAM #22b is formed by “bedforms that fluctuate in migration speed and asymmetry”. BAM #29 represents “simulated tidal bundles”. BAM #43a is formed by “bedforms with along-crest-migrating, out-of-phase sinuosities”. BAM #46n is formed by “bedforms with along-crest-migrating superimposed bedforms”. BAM #59 is formed by “migrating bedforms with spurs that reverse asymmetry and migration direction but have no net along-crest displacement”. BAM #67 is formed by “reversing, sinuous bedforms with reversing, superimposed, two-dimensional bedforms”. BAM #72 is formed by “straight-crested bedforms with superimposed, sinuous, out-of-phase bedforms migrating obliquely downslope”. For more information on the BAMs, we would suggest referring to Rubin and Carter^38^.

### Grain size distribution

**Figure 3:**
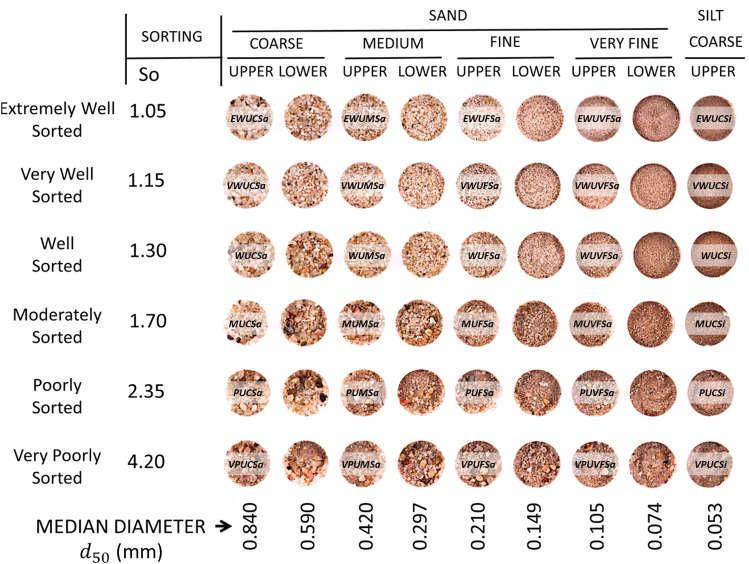
Grain size and grain sorting for unconsolidated sand mixtures^18,41,44^. ${d}_{50}$: The median grain size. ${S}_{0}$: Trask sorting coefficient. Full description is also labelled on some sand mixture pictures. Taking the top leftmost sand mixture as an example, EW-U-C-Sa means “Extremely Well Sorted Upper Coarse Sand”. Figure is adapted from Meckel et al.^39^.

In order to populate the BAMs with realistic petrophysical input parameters in the simulator, we assign lognormal grain size distributions based on experimental data to both the matrix and the laminae facies. By mixing unconsolidated sands into different categories of grain size and sorting^41^ as shown in Fig. 3, we can compute the mean and the standard deviation values of the lognormal grain size distributions using Eqs. (1) to (3),

1 $$\mu =\mathrm{ln}\left({d}_{50}\right)$$

2 $$\sigma =\frac{\mathrm{ln}\left({S}_{0}\right)}{0.6745}$$

3 $${S}_{0}=\sqrt{\frac{{d}_{75}}{{d}_{25}}}$$

where $\mu$ is the mean and $\sigma$ is the standard deviation of the resulting normal distribution after taking the natural log of the lognormal grain size distribution. ${S}_{0}$ is the Trask sorting coefficient^42,43^. $\sigma$ is determined by grain sorting. The more well sorted the grains are, the smaller the $\sigma$ in the grain size distribution. ${d}_{25}$, ${d}_{50}$, and ${d}_{75}$ are the 25th, 50th (median), and 75th percentile grain diameters in millimeters. 0.6745 is the constant associated with computing quartiles in a normal distribution.

After the grain size distributions for different types of sands are properly defined, we convert the lognormal grain size distributions into lognormal distributions of threshold capillary pressure, ${P}_{th}$, using Eqs. (4) to (6),

4 $${\mu }_{P}=-\mu +\mathrm{ln}\left(16.3\gamma \right)$$

5 $${\sigma }_{P}=\sigma$$

6 $${P}_{th}=16.3\times \frac{\gamma }{d}$$

where ${\mu }_{P}$ is the mean and ${\sigma }_{P}$ is the standard deviation of the normal $\mathrm{ln}({P}_{th})$ distribution transformed from the grain size distribution using Eq. (6)^45^. ${P}_{th}$ has a unit of kPa. d is the grain diameter in millimeters and $\gamma$ is the interfacial tension (IFT) between the CO_2_ and the water phase in N/m, taken to be 0.03 N/m to represent typical reservoir conditions^46^. 16.3 is the constant associated with pore geometries and unit conversion^45^. Finally, the mean (m.) and standard deviation (s.d.) of the lognormal distribution of ${P}_{th}$ can be computed with Eqs. (7) and (8). These two parameters are direct inputs into the MIP simulator.

7 $$m.={e}^{\left({\mu }_{P}+\frac{1}{2}{\sigma }_{P}^{2}\right)}$$

8 $$s.d.=m.\sqrt{{e}^{\left({\sigma }_{P}^{2}\right)}-1}$$

### Grain size contrast

Because natural sediment depositional processes cause the grains to segregate and form coarse-grained matrices and fine-grained laminae, it is necessary to assign each resulting ${P}_{th}$ distribution to the correct matrix and laminae facies in the corresponding BAM. 40 different matrix-laminae grain size contrast cases are selected to cover a wide range of heterogeneity values based on the following two criteria: (a) both the matrix and the laminae facies have the same sorting; (b) laminae have grain sizes less than or equal to that of the matrix.

The dimensionless grain size contrast parameter, $\delta$, is defined to gauge the degree of grain size (and the resulting ${P}_{th}$) heterogeneity in the domain. It is shown in Eq. (9)^22,37^,

9 $$\delta =\frac{\left|{\mu }_{1}-{\mu }_{2}\right|}{({\sigma }_{1}+{\sigma }_{2})/2}$$

where ${\mu }_{i}$ and ${\sigma }_{i}$ (i = 1, 2) can either be the mean and standard deviation respectively of the logged grain size distribution specified in Eqs. (1) and (2) or of the logged ${P}_{th}$ distribution specified in Eqs. (4) and (5). Subscripts 1 and 2 represent the matrix and the laminae facies. In this study, ${\sigma }_{1}$ and ${\sigma }_{2}$ are taken to be the same.

### Numerical model

All MIP simulations are conducted using Permedia®’s Static Migration module^47^. Permedia’s MIP simulator is based on the classical IP algorithm. The classical continuum-scale IP algorithm replicates the primary drainage process of a nonwetting phase fluid emitted from a point source by modelling a continual invasion of the neighboring grid block with the next lowest threshold capillary pressure (${P}_{th}$) value^28,29,48^. The ${P}_{th}$ values are determined by interfacial tension ($\gamma$) and grain size (d) as shown in Eq. (6). The driving potential ($\Phi$) for the migration of the nonwetting phase fluid is buoyancy ($\Delta \rho gz$) and hydrodynamic pressure (${\Phi }_{h}$) as shown in Eq. (10),

10 $$\Phi =\Delta \rho gz+{\Phi }_{h}$$

where $\Delta \rho$ is the density difference between the nonwetting phase and the wetting phase, g is the gravitational acceleration, and z is relative depth. To implement hydrodynamic pressure, the original ${P}_{th}$ field is modified to incorporate ${\Phi }_{h}$ prior to running the IP algorithm^49^. As for buoyancy, Permedia implements special migration and trapping rules to reflect previously known theoretical and lab findings.

Past studies have shown that under strongly capillary-dominated flow regimes (i.e. regimes with low flow rates), the nonwetting phase fluid migrates by pulsing and forms fragmented finger flow paths that have low saturation^45,50^. However, the nonwetting phase forms backfilled pools with high saturation when encountering baffles/capillary barriers^51,52^. Therefore, in Permedia’s MIP simulations, only the pools build up significant buoyancy pressure, while the fingers have negligible buoyancy pressure. The maximum nonwetting phase column height (h) within the pool is determined by Eq. (11),

11 $$h= \frac{{\Delta P}_{th}}{\Delta \rho g}$$

where $\Delta {P}_{th}$ is the difference in ${P}_{th}$ between the matrix and the laminae facies. As the nonwetting phase fluid migrates through the domain, the migration direction is determined by the driving potential gradient, and the nonwetting phase fluid pools behind capillary barriers until the maximum column height is reached before it breaks through.

Saturation-wise, any cells invaded by CO_2_ that are in the fingers will be assigned the fine-grid critical CO_2_ saturation value (${S}_{gcr}$) and those in the pools will be assigned the CO_2_ saturation value ($1-{S}_{wc}$; ${S}_{wc}$: irreducible water saturation)^18,47^. ${S}_{gcr}$ is the minimum CO_2_ saturation needed for the CO_2_ (nonwetting) phase to span the fine-grid cell. This is the CO_2_ saturation below which the CO_2_ phase is no longer continuous in the cell. $1-{S}_{wc}$ is the maximum CO_2_ saturation that the cell can contain. However, this is only the case at the field scale. For smaller scales such as the case in this study, all invaded cells are assigned the CO_2_ saturation value $1-{S}_{wc}$ to compensate for the small cell size. Therefore, the effective domain CO_2_ saturation output is dependent on the input value for ${S}_{wc}$. Because determining the exact value of ${S}_{wc}$ is out of the scope of the current study, we instead focus on the normalized CO_2_ saturation, $\langle {S}_{C{O}_{2}}\rangle$ (Eq. (12)), that is independent of the ${S}_{wc}$ value and is solely determined by the CO_2_ plume distribution in the domain.

12 $$\langle {S}_{C{O}_{2}}\rangle =\frac{{S}_{C{O}_{2}}}{1-{S}_{wc}}=\frac{cells\; invaded}{total\; cells}$$

### Simulation setup

All input parameters entered into the Permedia MIP simulator are listed in Table 1.

**Table 1:** Domain size, grid and fluid properties used in the MIP simulations.

	Input parameters	Input values
Domain size	Number of cells	101 × 101 × 101
	Cell size	2 mm × 2 mm × 2 mm
Grid property	Porosity,$\phi$	0.2
	Fine-grid critical CO_2_ saturation,${S}_{gcr}$	0.02
	Irreducible water saturation,${S}_{wc}$	0.2
	Threshold capillary pressure,${P}_{th}$	Lognormal(${\mu }_{P}$, ${\sigma }_{P}$)
Fluid model	CO_2_ density	700 kg/m^3^
	Water density	1000 kg/m^3^
	Interfacial tension	0.03 N/m

As shown in Table 1, the total domain size is 0.202 m × 0.202 m × 0.202 m with about ${10}^{6}$ cells. This domain size is selected based on the previous sensitivity analysis conducted by Trevisan et al.^18^. Results have shown that any subvolumes extracted from the BAMs that are above this size demonstrate consistent matrix-to-laminae ratios, which are equivalent to the net-to-gross sand/shale ratios^53^. The cell size is selected to be above the scale of the representative elementary volume so that petrophysical properties such as porosity and capillary pressure are well defined at the cell level^18,25,54,55^.

For both matrix and laminae facies, grid properties such as porosity ($\phi$), ${S}_{gcr}$, and ${S}_{wc}$ use Permedia default values and are kept constant across all cells. The only grid property that we vary across simulations is the ${P}_{th}$ field, which is derived using the method previously explained in Section "Grain size distribution". All grid properties for each facies are assumed to be isotropic in each cell. The bedform architecture creates natural anisotropy. The fluid model is selected based on typical CO_2_ geologic storage conditions and is also kept constant across simulations^18^. Simulation ${P}_{th}$ input data for all grain size contrast cases can be found in supplementary information.

The domain is initially assumed to be completely water filled. CO_2_ enters the domain through a planar source at the bottom and rises through the domain through buoyancy. The MIP simulation stops when CO_2_ percolates the top of the domain (percolation threshold). To ensure that simulation continues until the percolation threshold, the domain is set to have closed boundaries on all sides. For each of the 40 grain size contrast cases, 50 stochastic ${P}_{th}$ property field variations are generated by randomly drawing values from the pre-defined lognormal distributions.

## Results and discussion

### Effects of grain size contrast

**Figure 4:**
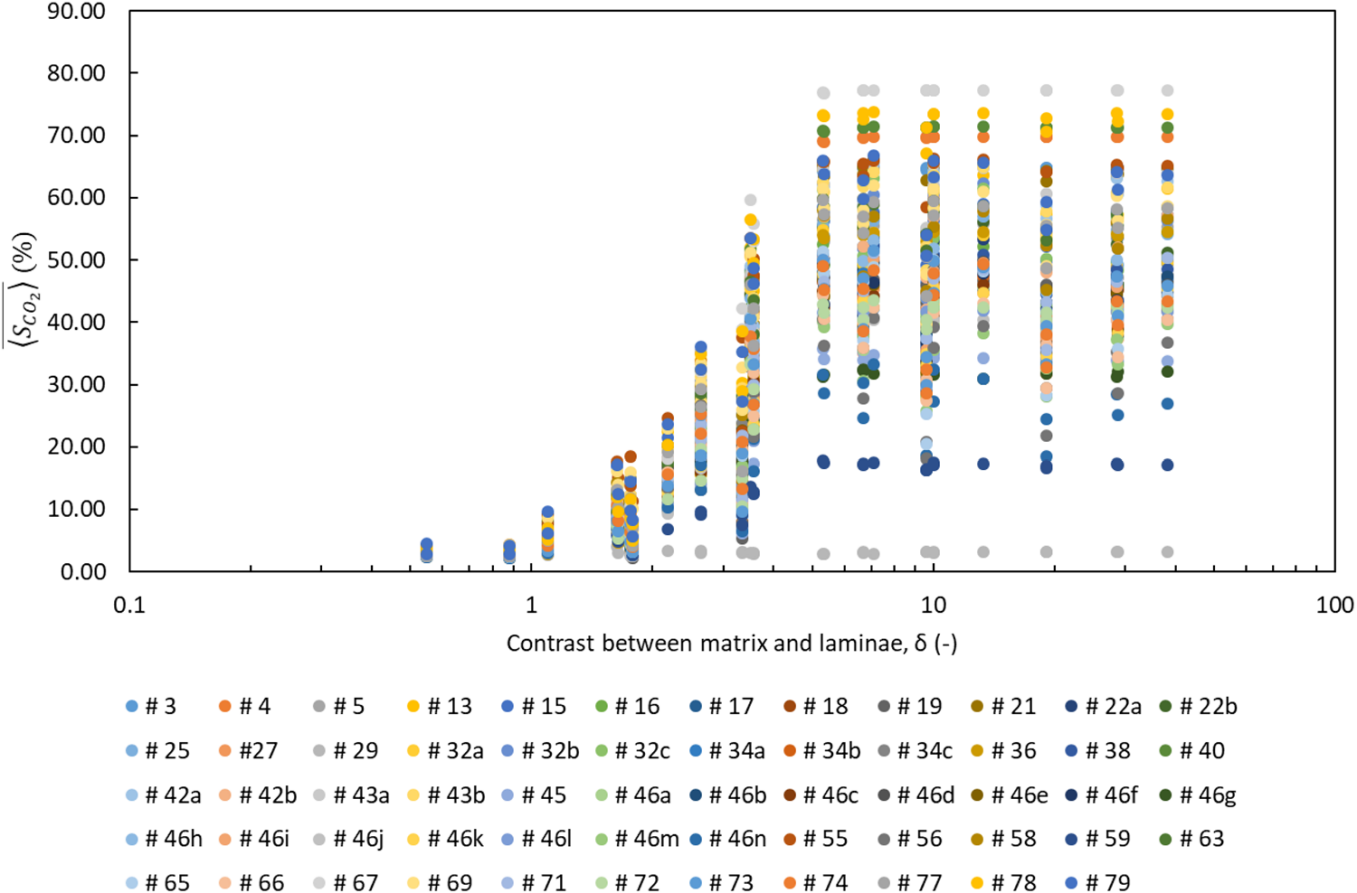
Mean domain CO_2_ saturation ($\overline{\langle {S }_{C{O}_{2}}\rangle }$) as a function of the dimensionless grain size contrast parameter (δ) for different BAMs. Every data point is the average of 50 stochastic simulation runs. The legend shows the color of the data series for the 59 BAMs and their corresponding number as defined by Rubin and Carter^38^.

As a result of having 59 BAMs, 40 grain size contrast cases, and 50 stochastic ${P}_{th}$ fields, a total of 118,000 simulation runs have been conducted. The mean domain CO_2_ saturation averaged over 50 stochastic simulation runs ($\overline{\langle {S }_{C{O}_{2}}\rangle }$) for the different BAMs and grain size contrasts can be visualized in Fig. 4. Numerical values for all simulation results can be found in supplementary information.

The results in Fig. 4 show that at low grain size contrast (a low degree of heterogeneity), CO_2_ saturation reached in the domain at the percolation threshold is low. As grain size contrast increases to reflect higher degrees of heterogeneity, CO_2_ saturation in the domain also increases to much higher levels, though not linearly. As a consequence, the data series for each BAM almost always displays a distinct “S” shape (Fig. 4). This result demonstrates the strong effect of grain size contrast on CO_2_ buoyant flow saturation and is consistent with the result from Trevisan et al.^18^. The grain size contrast between the matrix and the laminae facies directly translates to the threshold capillary pressure ratio of the two facies. Therefore, domain CO_2_ saturation increases with grain size contrast because greater laminae threshold capillary pressure promotes more CO_2_ column height buildup underneath each lamination layer. A maximum domain CO_2_ saturation value exists because as the laminae threshold capillary pressure increases, more CO_2_ backfilling occurs underneath the lamination layers and at some point all the matrix pore space available underneath the lamination layers is filled. Hence, further increase in the laminae threshold capillary pressure would no longer increase the domain CO_2_ saturation.

### Effects of bedform architecture

In Fig. 4, it is also observed that at low grain size contrast, different BAMs have similar $\overline{\langle {S }_{C{O}_{2}}\rangle }$ values, whereas at high grain size contrast, the values diverge. This result indicates that different bedform architectures only have a substantial influence on CO_2_ buoyant flow saturation when the domain has a high degree of heterogeneity.

**Figure 5:**
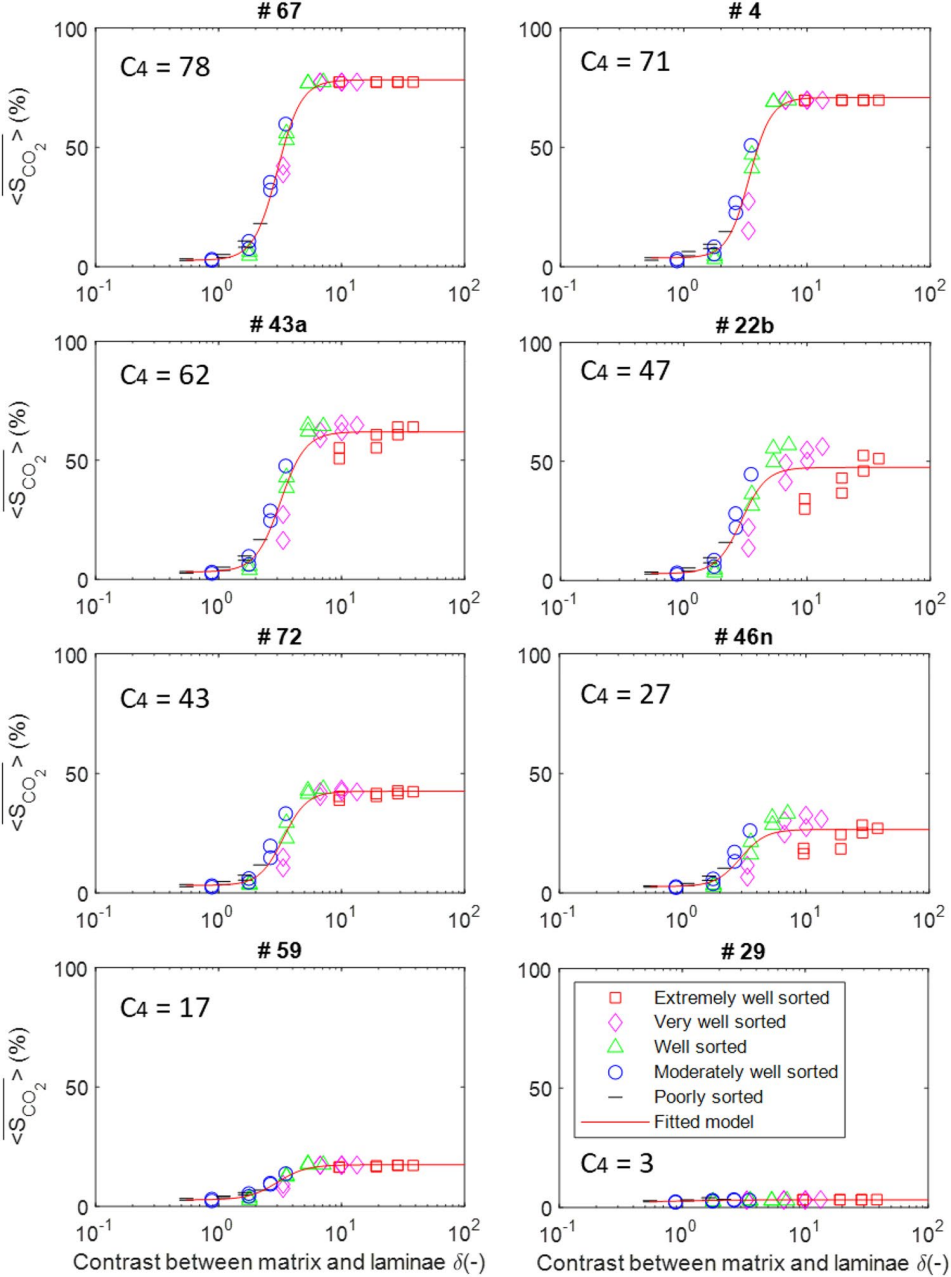
Mean domain CO_2_ saturation data and the corresponding fitted model for eight selected BAMs. The different symbols represent different grain sorting. The fitted model is shown with a red line. The value of the fitted parameter ${C}_{4}$ for each BAM is also displayed.

Trevisan et al.^18^ have devised a four-parameter equation to fit the S-shaped curve for each BAM, as shown in Eq. (11),

11 $$\overline{\langle {S }_{C{O}_{2}}\rangle }={C}_{4}+\frac{{C}_{1}-{C}_{4}}{1+{\left(\frac{\delta }{{C}_{3}}\right)}^{{C}_{2}}}$$

where ${C}_{1}$ and ${C}_{4}$ define the minimum and the maximum asymptotes at the two ends of the S curve, while ${C}_{2}$ and ${C}_{3}$ define the slope and the inflection point of the S curve. This equation fits the simulation results for all BAMs quite well, with a mean coefficient of determination (R^2^) value of 0.927. Fitted S curves for selected BAMs are shown in Fig. 5. Figure 5 shows that BAMs #59 and #29 have unusually low ${C}_{4}$ values, the reason for which is explained next.

**Figure 6:**
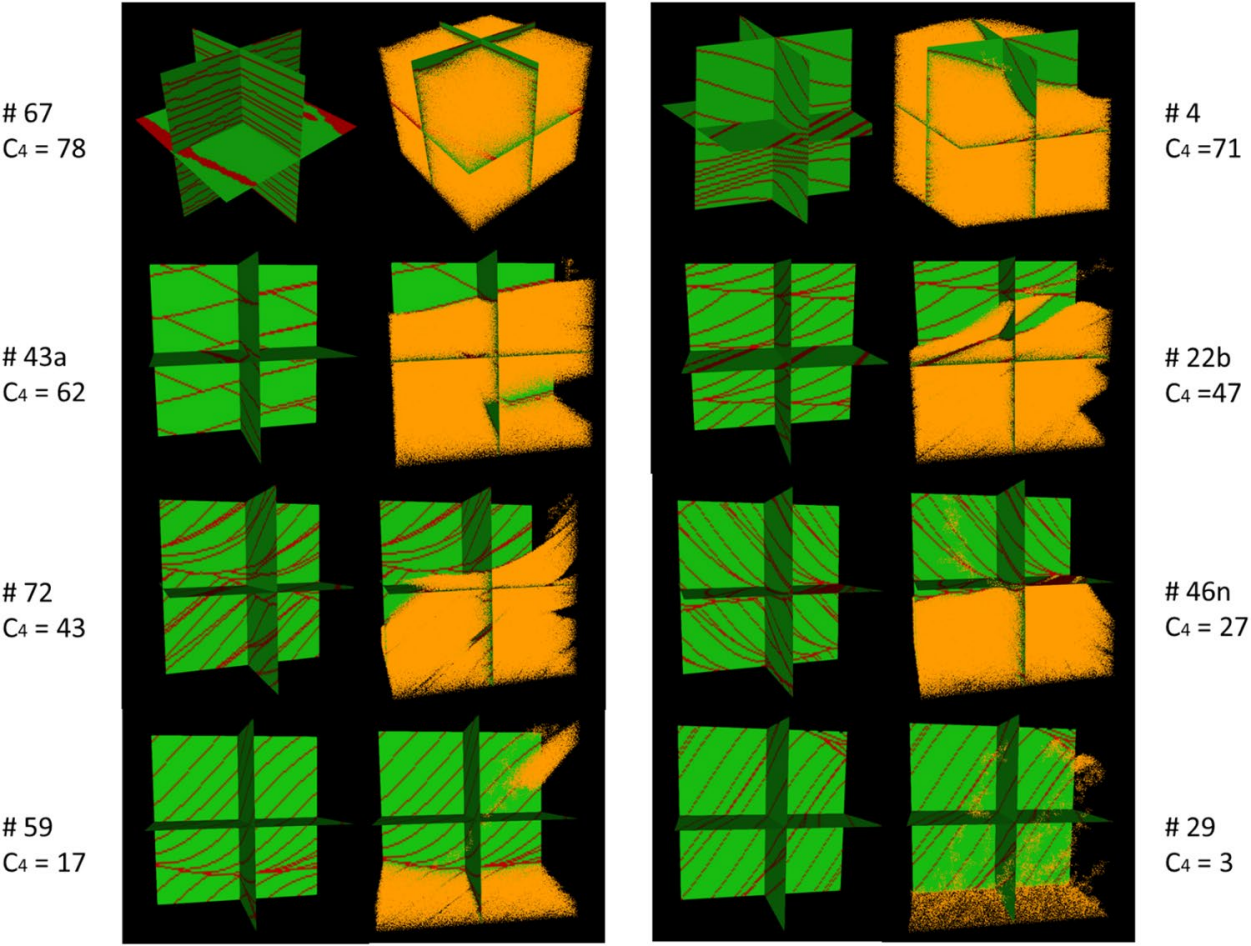
Bedform architecture and CO_2_ saturation distribution for eight selected BAMs. Green: matrix; red: laminae; orange: CO_2_ invaded cells with saturation $1-{S}_{wc}$. The CO_2_ saturation distribution is taken from a single simulation run with high grain size contrast values. The value of the fitted parameter ${C}_{4}$ for each BAM is also displayed.

Figure 6 shows the bedform architecture and the CO_2_ saturation distribution for the same set of BAMs featured in Fig. 5. From Fig. 6, we can see that at high grain size contrast, BAMs with continuous ripple lamination (#67 and #4) tend to trap more CO_2_, whereas BAMs with cross lamination (#43a, #22b, #72, and #46n) trap less CO_2_. Finally, BAMs with discontinuous cross lamination trap the least CO_2_. In Fig. 5, the two exceptional BAMs (#59 and #29) with much flatter curves instead of a typical S curve both have discontinuous cross lamination punctured with holes.

The reason that continuous ripple lamination retains more CO_2_ than discontinuous cross lamination at high grain size contrast is as follows. Because ripple lamination tends to be flatter than cross lamination, this allows CO_2_ to spread to a much greater area laterally before building up enough column height vertically to break through the lamination. Whereas for cross lamination, CO_2_ can easily rise along a cross lamina and break through without spreading laterally. Therefore, fine ripple lamination that is closely spaced allows for greater CO_2_ sweep of the domain during its buoyant migration upward. In addition, continuous lamination forces CO_2_ to build up column height underneath, whereas discontinuous lamination simply lets CO_2_ through so that it bypasses the laminae altogether. Therefore, discontinuous lamination barely retains any buoyant flow CO_2_.

### Effects of grain sorting

From Fig. 5, it can be clearly seen that for some BAMs (#43a, #22b, and #46n) the data points do not all collapse onto the fitted S curves as well as they do for other BAMs. It tends to be the case that BAMs with cross lamination (#43a, #22b, and #46n) have much greater spread around the fitted models than BAMs with ripple lamination (#67 and #4). This spread around the fitted curve is especially large for the “extremely well sorted” grain sorting type.

**Figure 7:**
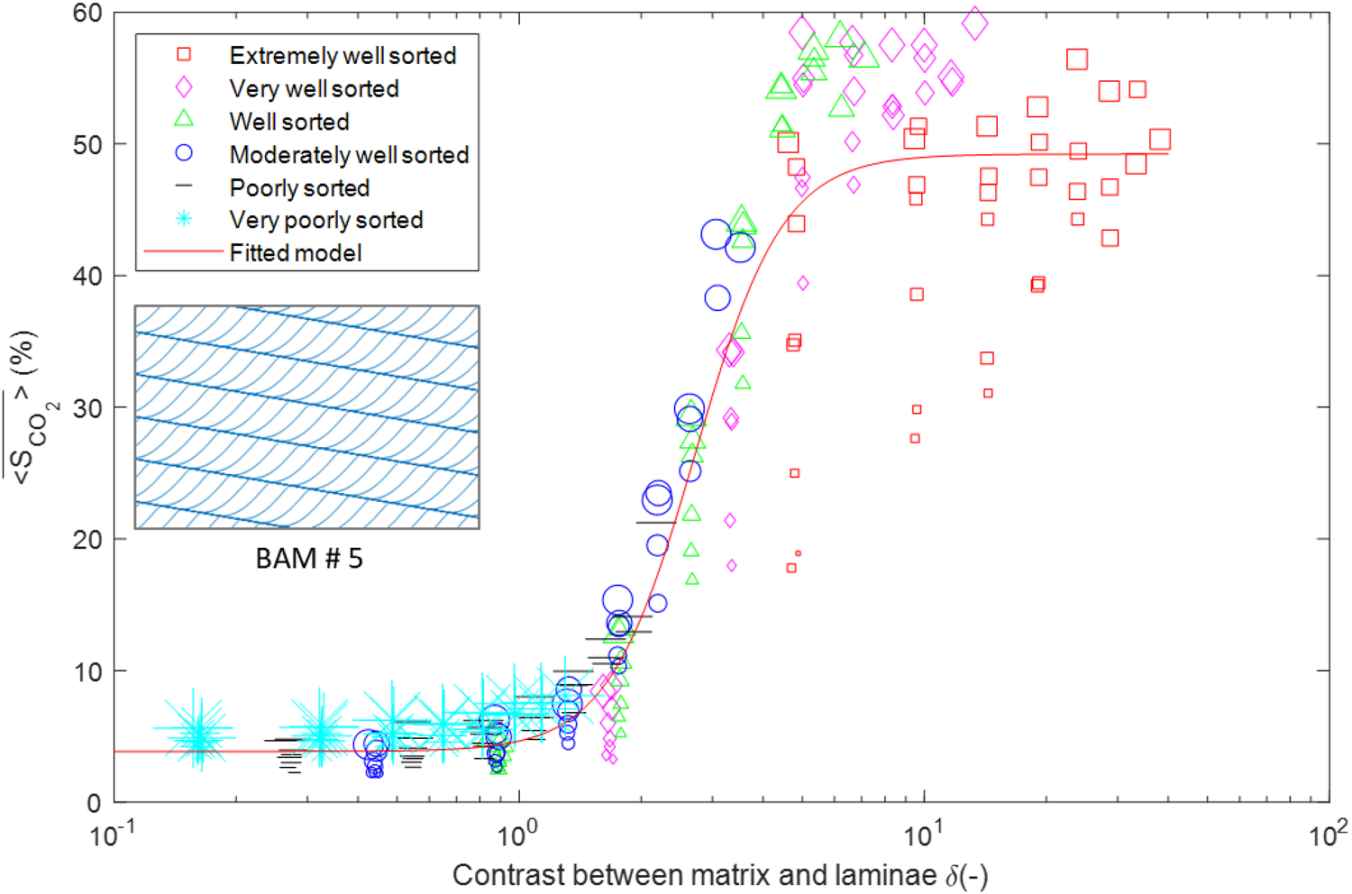
Mean domain CO_2_ saturation data and the fitted model for BAM #5 for all 270 grain size contrast cases. The inset graph shows the cross lamination pattern for BAM #5. The size of the symbols represents the laminae mean threshold capillary pressure (m. as defined in Eq. (7)). For more figure description, see Fig. 5.

To investigate the effect of grain sorting on CO_2_ buoyant flow saturation, we have conducted extra simulation runs on one particular BAM with 270 grain size contrast cases. The BAM selected is #5 as the simulation data points have a discernable spread around the fitted model. The 270 grain size contrast cases are those that satisfy the selection criteria in Section "Grain size distribution". To reduce computational intensity, only 10 stochastic simulation runs are conducted per grain size contrast case. The simulation results are shown in Fig. 7. The fitted model (solid red line) has highly similar parameters as those of the model fitted on just 40 grain size contrast cases, indicating that having 40 cases is sufficient in capturing the full range of CO_2_ buoyant flow saturation values.

As shown in Fig. 7, it is clear that while at low grain size contrast all the data points collapse well onto the fitted model, at high grain size contrast there is a much greater spread in the values. This is especially the case for the “extremely well sorted” grain sorting type. A closer examination of the size of the symbols (mean ${P}_{th}$ for the laminae facies) in Fig. 7 shows that at the same δ value, domains with greater laminae ${P}_{th}$ (finer laminae) tend to retain more CO_2_. This is true regardless of the grain sorting type, but the effect is especially pronounced within the “extremely well sorted” category, which has some particularly small laminae ${P}_{th}$ values. Cross-laminated domains with small laminae ${P}_{th}$ values tend to retain less CO_2_ than the fitted model compared to ripple-laminated domains because the existence of cross-lamination tends to favor upward migration and hinder lateral spreading of the plume.

### Effects of the fluid model

**Figure 8:**
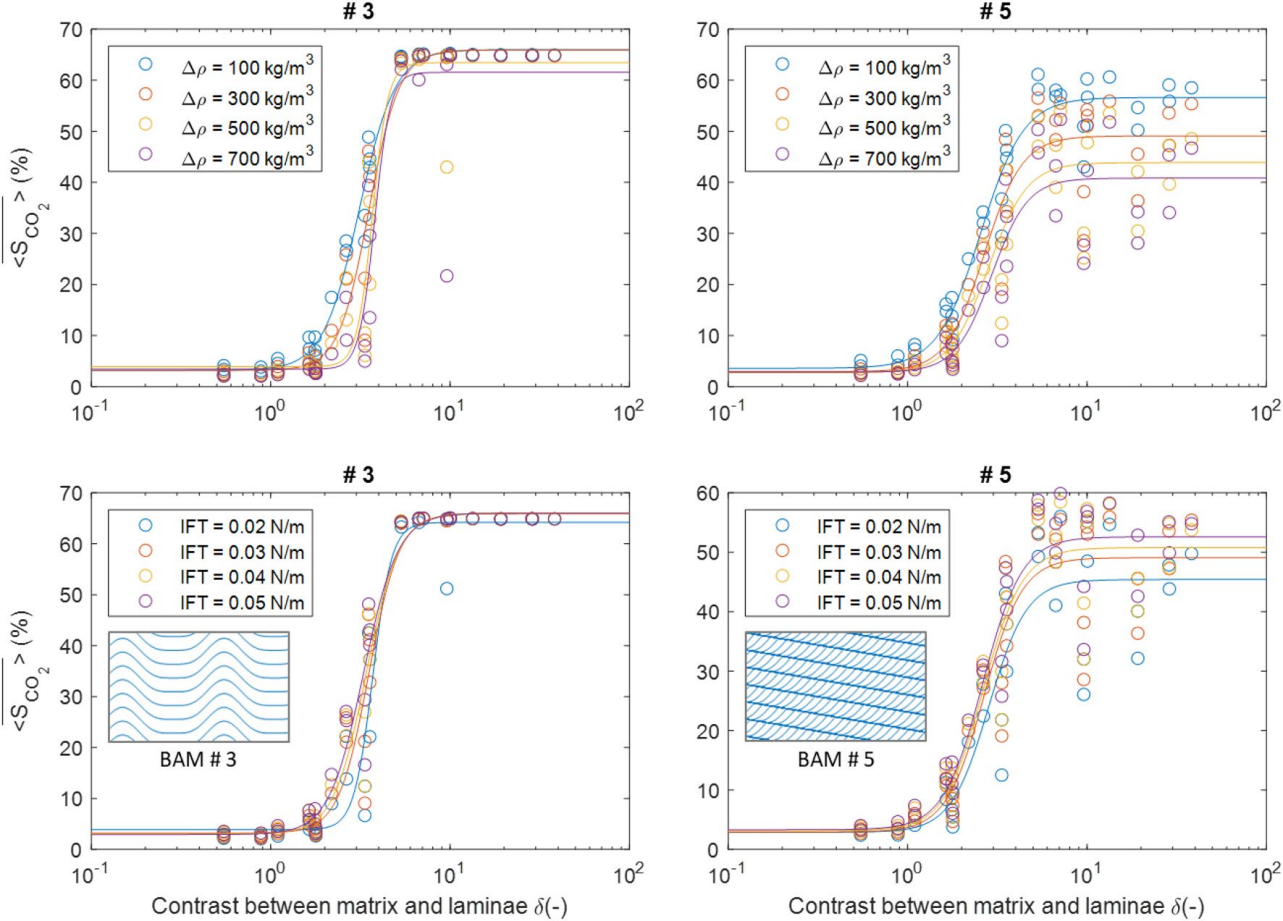
Effects of varying the density difference and the IFT value for two selected BAMs. The inset graphs show the BAM patterns. The fitted models have solid lines with the same color as the data series shown in circles.

Further simulations on eight selected BAMs have been conducted to investigate the effect of differing density contrast and IFT values on CO_2_ buoyant flow saturation. The base case fluid model has a density contrast of 300 kg/m^3^ and an IFT value of 0.03 N/m between the CO_2_ and the water phase. In this section, new values for the fluid model are used: (a) density difference: 100, 500, and 700 kg/m^3^; (b) IFT: 0.02, 0.04, and 0.05 N/m. Each fluid property is changed individually while keeping the other input parameters the same as the base case. To obtain the desired density difference, the water density is kept constant while only the CO_2_ density is reduced. Selected results are shown in Fig. 8.

As shown in Fig. 8, while increasing the density difference decreases CO_2_ buoyant flow saturation, increasing the IFT value increases CO_2_ saturation. This is because a less buoyant nonwetting phase fluid can build up more column height before breaking through the capillary barrier. A higher IFT value means stronger capillary forces opposing CO_2_ breakthrough, which also allows for more column height buildup. Larger column height means greater rock volume invaded beneath the capillary barrier, and directly translates to higher CO_2_ saturation in the domain. It is also generally the case that the CO_2_ saturation of ripple-laminated domains is less affected than cross-laminated domains by changes in the density difference and IFT at high grain size contrast. This is because the existence of cross lamination compartmentalizes the domain, increasing the number of possible migration paths that the CO_2_ can take before it breaks through the domain at the top.

### Uncertainty and verification

Previously, it has been difficult to quantify the effect of small-scale heterogeneity on CO_2_ LCT for buoyancy-driven flow because of the complexity of such heterogeneities and lack of data. However, as shown by the simulation results, the impact of small-scale heterogeneity on critical CO_2_ saturation is significant. Depending on the types and degrees of the heterogeneity, the resulting critical CO_2_ saturation value can vary between 2 and 77%. As explained in the introduction, such a wide range of critical CO_2_ saturation values can greatly affect the field-scale CO_2_ trapping capacity^23,36^. Therefore, it is of interest to develop prediction models that can accurately quantify the influence of small-scale heterogeneity. The value of this study is then to provide a comprehensive simulation dataset, upon which said prediction models can be built for upscaling purposes in field-scale simulations.

Because the reported simulated critical CO_2_ saturation values are averaged over 50 stochastic runs, the associated uncertainty can be represented by the standard deviation across the stochastic runs. The standard deviation values associated with each bedform and grain size contrast case are generally small, with a mean value of 3% and a maximum value of 12%. In this study, only vertical flow simulations are conducted, and the resulting domain effective critical CO_2_ saturation values can be treated as isotropic. However, this will only be accurate if the grain size contrast is low. At high grain size contrast, the trapping amount tends to be different if horizontal flow simulation is also conducted. This is particularly likely to be the case for bedforms with higher degrees of anisotropy, or ripple bedforms. Therefore, future simulation studies should focus on further simulations to quantify the effect of anisotropy on critical CO_2_ saturation. For any critical CO_2_ saturation prediction model built upon this simulation dataset, another source of uncertainty would be the effect of the fluid properties. The assumption is that typical geologic CO_2_ storage conditions should be similar to the simulation setup in this study. And as Fig. 8 shows, slight deviations in density contrast or IFT from the base case values should not lead to major differences in critical CO_2_ saturation.

In order to verify that the simulation results are reliable and accurate, we compare the simulation results to previous experimental results^22,37,56^. These are physical fluid flow experiments conducted in Hele-Shaw cell type sand tanks with analog fluids with similar fluid properties to this study and at low flow rates so that the flow regime is strongly buoyancy- and capillary-dominated as is the case for the simulation. Realistic sedimentary bedforms were packed in the sand tank with different grain size contrast cases. Not only do the experimental results demonstrate the same S-curve with increasing grain size contrast, but the critical CO_2_ saturation values also roughly match the simulation values for the specific BAM #5^37^. In the future, more physical experiments should be conducted to verify the simulation results for the other BAMs.

Because the domain effective critical CO_2_ saturation values in this study are obtained at vanishing flow rates with no viscous forces, one concern may be how the addition of viscous forces would affect the upscaling of this parameter. At low degrees of heterogeneity, we would likely have a viscous fingering pattern with early breakthrough at high flow rates, which would lead to lower critical CO_2_ saturation values^57,58^. However, at high degrees of heterogeneity, higher viscous forces (greater injection rates) would increase the critical CO_2_ saturation at domain percolation, according to another set of physical experimental results for BAM #5^22^. Therefore, the interplay between heterogeneity and flow rates determines the domain effective critical CO_2_ saturation values.

## Conclusion

To investigate how grain size and bedform architecture affect CO_2_ buoyant flow saturation, we ran 118,000 MIP simulations, covering 59 BAMs, 40 grain size contrast cases, and 50 stochastic variations. Simulation results show that grain size contrast has a considerable impact on the effective CO_2_ saturation for heterogeneous domains, whereas bedform architecture only becomes important at high grain size contrast values. Different grain sorting as well as varying density differences and IFT values also affect the simulated domain effective CO_2_ saturation. More specifically, the following conclusions can be reached.

1. The domain effective CO_2_ buoyant flow saturation value increases nonlinearly with increasing grain size contrast values. The relationship can be described by a parametrized S-shaped curve for bedform architectures with continuous lamination layers.
2. At low grain size contrast values, effective CO_2_ saturation values for different BAMs all have similar values. However, as grain size contrast values increase, different BAMs reach different maximum CO_2_ saturation values. Domains with continuous ripple lamination tend to have greater maximum CO_2_ saturation values than domains with discontinuous cross lamination.
3. Simulations of the “extremely well sorted” grain sorting type often tend to deviate from the S-shaped curve for domains with cross lamination. At the same grain size contrast value, domains with finer lamination grains tend to retain more CO_2_.
4. Domain effective CO_2_ saturation decreases with increasing density differences between the CO_2_ and the water phase, and increases with increasing IFT values between the two phases. The strength of the influence that the fluid model has on CO_2_ saturation is bedform dependent.

## Supplementary Information

Supplementary Information.

## Data Availability

All simulation input and output data is available in supplementary information.
